# Supplementary material for: Diagnostics and Management of Pediatric Headache: An Exploratory Study among Dutch Physical Therapists
Source: Children (Basel). 2023 Jun 30;10(7):1135. doi: 10.3390/children10071135 (PMC10378271; doi:10.3390/children10071135)
Supplement: Supplementary file 1 [file children-10-01135-s001.zip › File S1.pdf]

Questionnaire headache among children up to 18 years

The questionnaire was intended to gather various options, opinions, advice, and thoughts regarding three clinical domains: 1. diagnostic screening (anamnestic items, questionnaires), 2. physical therapeutic clinical examination, and 3. physical therapeutic management.

Demographic data

Gender	☐ male	☐ female
Age	……… year	
Nationality	☐ Dutch	☐ different;……………………
Highest education	☐ BSc ☐ MSc ☐ PhD	
Profession	………	
Specialisation	………	
Working setting	☐ first-line practice	
	☐ health centre	
	☐ general hospital	
	☐ university hospital	
	☐ rehabilitation centre	
	☐ otherwise;	

Estimated number of patients with headaches per month ....

Estimated number of children with headaches per month ....

Estimated number of new children with headaches per month ....

Do you use the IHS criteria to classify headaches? Yes / no

if not, what classification do you use?: ....

1. Diagnostic screening (a. anamnestic items, b. questionnaires)

a. Indicate how useful you find each anamnestic item during diagnostic screening to classify headaches in children.

Classification of headache	Very useful	useful	Don't know	Probably not useful	Certainly not useful
**Diagnostic screening; anamnestic items**					
Severity of headache	☐	☐	☐	☐	☐
Location of headache	☐	☐	☐	☐	☐
Type of headache (pounding, pulling, pressing, shooting,...)	☐	☐	☐	☐	☐
Duration of headache	☐	☐	☐	☐	☐
Headache time of day	☐	☐	☐	☐	☐
Patient report about the course of headache	☐	☐	☐	☐	☐
Headache frequency	☐	☐	☐	☐	☐
Headache increase duet to activities	☐	☐	☐	☐	☐
photophobia	☐	☐	☐	☐	☐
Phonophobia	☐	☐	☐	☐	☐
Nausea	☐	☐	☐	☐	☐
Vomit	☐	☐	☐	☐	☐
Intoxications	☐	☐	☐	☐	☐
Previously established classification	☐	☐	☐	☐	☐
Family history of headache	☐	☐	☐	☐	☐

Classification of headache	Very useful	useful	Don't know	Probably not useful	Certainly not useful
**Diagnostic screening; anamnestic items**					

Stress	☐	☐	☐	☐	☐
School achievements	☐	☐	☐	☐	☐
High working pressure	☐	☐	☐	☐	☐
Social problems	☐	☐	☐	☐	☐
Tension at home	☐	☐	☐	☐	☐
Nxiety and panic attacks	☐	☐	☐	☐	☐
Self-reported depression	☐	☐	☐	☐	☐
Allergies	☐	☐	☐	☐	☐
Cold complaints	☐	☐	☐	☐	☐
Vision Complaints	☐	☐	☐	☐	☐
Aura complaints	☐	☐	☐	☐	☐
Moment and duration of aura	☐	☐	☐	☐	☐
Temporomandibular dysfunction	☐	☐	☐	☐	☐
Pain/sensitivity teeth	☐	☐	☐	☐	☐
Grinding/ clenching	☐	☐	☐	☐	☐
Sleep quantity	☐	☐	☐	☐	☐
Sleep quality	☐	☐	☐	☐	☐
Other (chronic) complaints	☐	☐	☐	☐	☐
Trigger/ maintaining factors	☐	☐	☐	☐	☐

b. Indicate how useful you find a headache diary and questionnaires as an addition to the diagnostic screening to classify headaches in children.

Classification of headache	Very useful	useful	Don't know	Probably not useful	Certainly not useful
**Diagnostic screening (questionnaires)**					
Headache diary:<br>At what time(s) of the day should it be filled in?:<br>..<br>How long should it be kept?:<br>……………………………………………………………………………………	☐	☐	☐	☐	☐
Questionnaires: (including headache-specific, physical activities, quality of life, pain coping, anxiety and panic, depression and sleep)<br>Which one?:……………………………………………………………………….<br>……………………………………………………………………………………<br>……………………………………………………………………………………<br>……………………………………………………………………………………<br>……………………………………………………………………………………<br>……………………………………………………………………………………<br>……………………………………………………………………………………<br>……………………………………………………………………………………<br>……………………………………………………………………………………<br>……………………………………………………………………………………<br>……………………………………………………………………………………<br>……………………………………………………………………………………	☐	☐	☐	☐	☐

2. Physical therapeutic clinical examination

Indicate how useful you find each item during physical therapeutic clinical examination to classify headaches in children.

Classification of headache	**Very useful**	**useful**	**Don't know**	**Probably not useful**	**Certainly not useful**
**Physical therapeutic clinical examination**					
Neurologic examination	☐	☐	☐	☐	☐
Measuring bloodpressure	☐	☐	☐	☐	☐
BMI (body mass index)	☐	☐	☐	☐	☐
Head size	☐	☐	☐	☐	☐
Body posture	☐	☐	☐	☐	☐
Head posture	☐	☐	☐	☐	☐
Changes, redness and swelling	☐	☐	☐	☐	☐
Movement examination neck	☐	☐	☐	☐	☐
Movement examination jaw	☐	☐	☐	☐	☐
Muscle palpation	☐	☐	☐	☐	☐
Triggerpoints	☐	☐	☐	☐	☐
Intraorale inspection	☐	☐	☐	☐	☐
Provoke headache by physical examination	☐	☐	☐	☐	☐
Provoke other complaints by physical examination	☐	☐	☐	☐	☐
Other physical examination tests?:…………………………………………………	☐	☐	☐	☐	☐

3. Physical therapeutic management

For each physical therapeutic management item, indicate a score for its potential usefulness in treating children with headaches. In other words, do you expect to obtain a positive result with this intervention in reducing headaches and / or the consequences of this?

Physical therapeutic management	**Very useful**	**useful**	**Don't know**	**Probably not useful**	**Certainly not useful**
**(in alphabetical order)**					
Biofeedback	☐	☐	☐	☐	☐
Cognitive behavioral therapy	☐	☐	☐	☐	☐
Dry-needling	☐	☐	☐	☐	☐
Habit reversal training	☐	☐	☐	☐	☐
Headache education	☐	☐	☐	☐	☐
Posture treatment	☐	☐	☐	☐	☐
Hypnose	☐	☐	☐	☐	☐
Lifestyle coaching	☐	☐	☐	☐	☐
Manipulation neck	☐	☐	☐	☐	☐
Massage and stretching	☐	☐	☐	☐	☐
Mobilisation Neck	☐	☐	☐	☐	☐
Meditation	☐	☐	☐	☐	☐
Occlusal appliances	☐	☐	☐	☐	☐
Exercise treatment by non-physical therapists	☐	☐	☐	☐	☐
Exercise treatment by physical therapists	☐	☐	☐	☐	☐
Pain education	☐	☐	☐	☐	☐

Physical therapeutic management	Very useful	useful	Don't know	Probably not useful	Certainly not useful
**(in alphabetical order)**					
Relaxation treatment	☐	☐	☐	☐	☐
Slaaphygiene	☐	☐	☐	☐	☐
Selfmonitoring	☐	☐	☐	☐	☐
Complementairy treatments:<br>………………………………………………………………………………………………<br>………………………………………………………………………………………………	☐	☐	☐	☐	☐
Other treatment interventions in children with headaches, which have not yet been mentioned?:<br>………………………………………………………………………………………………<br>………………………………………………………………………………………………	☐	☐	☐	☐	☐
