# Supplementary material for: Diagnostics and Management of Pediatric Headache: An Exploratory Study among Dutch Physical Therapists
Source: Children (Basel). 2023 Jun 30;10(7):1135. doi: 10.3390/children10071135 (PMC10378271; doi:10.3390/children10071135)
Supplement: Supplementary file 1 [file children-10-01135-s001.zip › File S2.pdf]

Appendix B - 4P-factor model [20-22]

A biopsychosocial approach and examples of what could be found for each domain and how to implement this approach in practice

	Biological factors	Psychological factors	Social factors
**Predisposing factors**	Genetic vulnerability<br>Family medical history<br>Birth defects<br>Developmental delays<br>Age, race, sex, gender	Personality traits<br>Temperament<br>Isolation, Insecurities, Fear<br>Distress tolerance	Adverse childhood experiences<br>Unstable home or school life<br>Chronic stress
**Precipitating factors**	Onset of illness or medical disorder<br>Poor sleep<br>Physical trauma<br>Substance use or misuse	Avoidant coping style<br>Poor problem solving<br>Negative or maladaptive thoughts<br>Psychopathology	Stressful life events<br>Social support, loss of significant relationship<br>Interpersonal conflict
**Perpetuating factors**	Substance use or misuse<br>Chronic illness/pain<br>Immunosuppression	Compensatory or avoidance behaviors<br>Inadequate underlying beliefs of self, others and the world	Overly busy school/life schedule<br>Treatment or social dilemma's<br>Poor finance
**Protecting factors**	Adequate diet, sleep, physical exercise<br>Good genes<br>Resilience<br>Intelligence	Adaptive coping mechanisms<br>Adequate behavior strategies<br>Ability to be reflective	Positive relationships<br>Social support and faith<br>Financial support<br>Practitioner's support

An example of how the 4P-factor model could be used to construct, formulate and generate a patient-oriented multidisciplinary treatment plan is set out below:

1. Brief summarize the headache complaints and presenting problems from child and parents perspective of signs and symptoms by using SOCRATES (Site, Onset, Character, Radiation, Associated Symptoms, Time course, Exacerbating or relieving factors, Severity). Also add diagnostic findings; physical examination and questionnaires.
2. Explore the child and parents ideas, concerns and expectations and bring together themes important to the child and family's perspective of functioning and well-being at home, school, and in the community by introducing the F-words (function, family, fitness, fun, friends, and future) [30,31]. Tools and resources are freely available on CanChild's F-words Knowledge Hub (CanChild, 2019)www.canchild.ca/f-words.
3. Summarise to check your understanding and provide an opportunity for the child and parents to correct any inaccurate or overlooked information.
4. Bidirectional exchange 4P-factor model and describe how the factors interact with the headache complaints, level of functioning, prognosis, and what the potential openings are for intervention.
5. Help to make choices of the most suitable combinations of treatment options, based also on the child and parents personal values and preferences. It may help to lay focus on what the child and parents 'can do' rather than 'cannot do' by using the F-words.
6. Evaluation of headache complaints over time together with the child and parents, and if necessary discuss possible further diagnostic and/ or (self-) management steps.
